# Cell membrane coated smart two-dimensional supraparticle for *in vivo* homotypic cancer targeting and enhanced combinational theranostics

**DOI:** 10.7150/ntno.57657

**Published:** 2021-02-14

**Authors:** Di Zhang, Zhongju Ye, Hua Liu, Xin Wang, Jianhao Hua, Yunyun Ling, Lin Wei, Yunsheng Xia, Shaokai Sun, Lehui Xiao

**Affiliations:** 1 College of Chemistry, Zhengzhou University, Zhengzhou, 450001, China.; 2 State Key Laboratory of Medicinal Chemical Biology, Tianjin Key Laboratory of Biosensing and Molecular Recognition, College of Chemistry, Nankai University, Tianjin, 300071, China.; 3 Key Laboratory of Functional Molecular Solids, Ministry of Education, College of Chemistry and Materials Science, Anhui Normal University, Wuhu, 241000, China.; 4 College of Chemistry and Chemical Engineering, Hunan Normal University, Changsha, 410081, China.; 5 School of Medical Imaging, Tianjin Medical University, Tianjin, 300071, China.

**Keywords:** cancer cell membrane, homotypic targeting, dual-modal imaging, gold nanorod, photothermal therapy.

## Abstract

Development of intelligent and multifunctional nanoparticle for the diagnosis and treatment of cancer has drawn great attention recently. In this work, we design a smart two-dimensional (2D) supraparticle for tumor targeted magnetic resonance imaging (MRI)/photothermal imaging (PTI) and chemo/photothermal therapy (PTT).

**Methods:** The nanoparticle consists of a manganese dioxide (MnO_2_) nanosheet coated gold nanorod (GNR) core (loading with chemotherapeutics doxorubicin (DOX)), and cancer cell membrane shell (denoted as CM-DOX-GMNPs). Decoration of cell membrane endows the nanoparticle with greatly improved colloidal stability and homotypic cancer cell targeting ability. Once the nanoparticles enter tumor cells, MnO_2_ nanosheets can be etched to Mn^2+^ by glutathione (GSH) and acidic hydrogen peroxide (H_2_O_2_) in the cytosol, leading to the release of DOX. Meanwhile, stimuli dependent releasing of Mn^2+^ can act as MRI contrast agent for tumor diagnosis. Illumination with near-infrared (NIR) light, photothermal conversion effect of GNRs can be activated for synergistic cancer therapy.

**Results:** *In vivo* results illustrate that the CM-DOX-GMNPs display tumor specific MRI/PTI ability and excellent inhibition effect on tumor growth.

**Conclusion:** This bioinspired nanoparticle presents an effective and intelligent approach for tumor imaging and therapy, affording valuable guidance for the rational design of robust theranostics nanoplatform.

## Introduction

According to the statistics from the international agency for research on cancer, there are about 14 million new cancer patients and 8 million people die from cancer-related diseases worldwide each year 1. The number of new cancer cases per year is expected to rise to 23.6 million by 2030. Owing to the high risk and death rate of cancer, great efforts have been paid to develop accurate, efficient and rapid theranostic strategies for cancer treatment 2, 3. At present, chemotherapy remains one of the main approaches for cancer treatment 4, 5. However, chemo-therapeutic drugs are easily causing systemic side effects and inducing complications to patients because of the lack of specificity for cancer cells and their serious toxicity to normal cells and tissues 6-8. In this regard, some new methods have been utilized to develop synergistic therapeutic platforms with chemotherapy in order to improve those deficiencies. Among those various strategies, photothermal therapy (PTT) is one of the most popular candidates, which can convert energy from light into heat and cause irreversible cell damage by using appropriate photothermal transduction agents (PTAs) and harmless near-infrared (NIR) laser 9-12. Compared with conventional therapeutic methods, PTT not only eradicates cancer cells in the primary tumor directly, but also produces specific lethal effect on the cells in the metastatic sites 10. Besides, the method displays superior specificity, minimal invasiveness and high efficiency in cancer therapy 1, 13, 14. More importantly, photothermal imaging (PTI) capacity along with PTT can also provide the possibility for real-time biomolecule tracking and disease diagnosis in organisms.

The most commonly used PTAs include inorganic and organic materials. In contrast to organic materials, inorganic PTA nanostructures have higher photothermal conversion efficiency and better photothermal stability. More interestingly, owing to the flexibility of the nanostructure, it is possible to integrate multiple imaging modalities and therapeutic functions into one compact nanostructure for intelligent and synergistic treatment. Among those inorganic PTAs, gold nanorods (GNRs) are increasingly used because of the strong absorption for heat conversion in the NIR region, versatile surface chemistry for biomolecule conjugation and outstanding biocompatibility over other inorganic nanostructures 15-18. Despite these attractive merits, using singlet PTT with GNRs as the PTA for cancer therapy still faces some grand challenges which have been criticized by researchers. In detail, one of the essential challenges is the limited depth of light penetration, which may lead to incomplete ablation of tumor outside the scope of irradiation. Besides, overheating of the tumor area might cause unnecessary damage to normal tissues, and development of resistances to PTT due to the overexpression of heat shock proteins in certain cancers. Integration of chemotherapy with PTT can greatly overcome the constraints as noted above and improve the treatment outcomes 19-21. However, owing to the limited drug loading efficiency, it is still a big challenge to apply GNRs for the efficient synergistic cancer treatment with PTT and chemotherapy.

In addition, poor tumor targeting ability is another substantial challenging issue for cancer nanotheranotics 12, 22. Although the size effect of the therapeutic agents can make them passively target to tumor site via the enhanced permeability and retention (EPR) effect, the accumulation efficiency is largely influenced by the tumor condition, including vascularization and permeability 23, 24. The method of modifying tumor-targeting molecules, such as aptamers and antibodies 25, 26, on the surfaces of therapeutic agents is also restricted by the complicated fabrication process, organic repulsion as well as recognition selectivity 22, 25-27. The development of cancer cell membrane coating strategy provides an alternative route for the design of new tumor targeting platform. It has been well-documented that the proteins and molecules on the cell surface are highly associated with the recognition and interaction between cancer cells 28, 29. By coating the nano-vehicles with cancer cell membrane, the biomimetic nanostructures are endowed with increasing biocompatibility and tumor-targeting property 29-31.

**Scheme 1:**
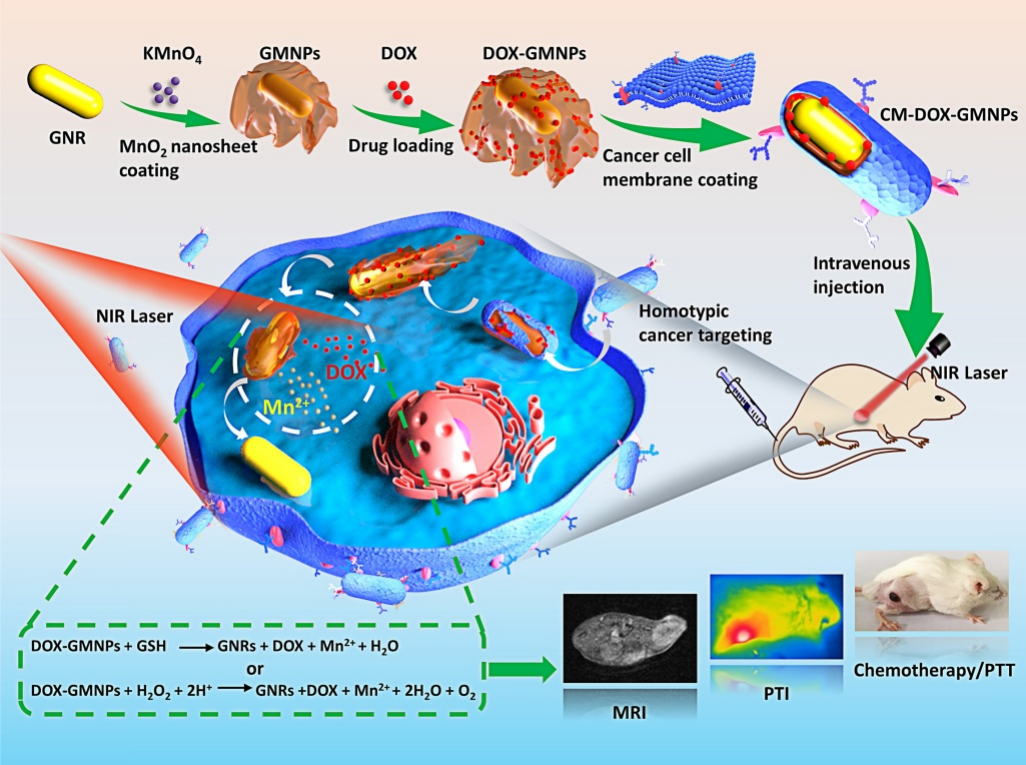
Schematic illustration of CM-DOX-GMNPs for tumor targeted magnetic resonance imaging (MRI)/photothermal imaging (PTI) and chemo/photothermal therapy (PTT).

Herein, an intelligent and compact two-dimensional (2D) heterostructure is designed for the tumor targeted photothermal and magnetic resonance dual modality imaging and chemo/photothermal therapy (Scheme 1). Specifically, 2D manganese dioxide (MnO_2_) nanosheets with large surface area are first grown around GNRs (GMNPs) and loaded with chemotherapeutic ingredients (*i.e.*, doxorubicin (DOX)) to form the core of the nanodrug (DOX-GMNPs). The biodegradable, flexible and ultrathin structure of the MnO_2_ nanosheet enables a stable and high drug loading efficiency for stimuli-dependent drug release. Meanwhile, introducing cancer cell membrane onto the surface of the nanostructure endows the particle with distinguished homotypic cancer cell targeting ability and excellent colloidal stability in drug delivery and endocytosis process. Once entering the cell, owing to the characteristic feature of the endogenous tumor microenvironment (TME), the membrane can be disrupted and the compact MnO_2_ nanosheets are etched to Mn^2+^ by endogenous glutathione (GSH) and acidic H_2_O_2_. This intelligent self-driven drug-releasing behavior under endogenous TME stimuli greatly alleviates the side effect caused by chemotherapy and also remarkably enhances the PTT efficacy from GNRs. Importantly, the released Mn^2+^ within the target tumor cells affords a robust platform for imaging-guided PTT through Mn^2+^-based magnetic resonance imaging (MRI). In contrast to the commonly used remedies, this self-driven intelligent theranostic nanostructure provides remarkably improved therapeutic efficacy for *in vivo* cancer treatment and also affords new insight into the development robust nanoplatform for imaging-guided synergistic cancer therapy.

## Results and discussion

### Preparation and characterization of GMNPs

**Figure 1:**
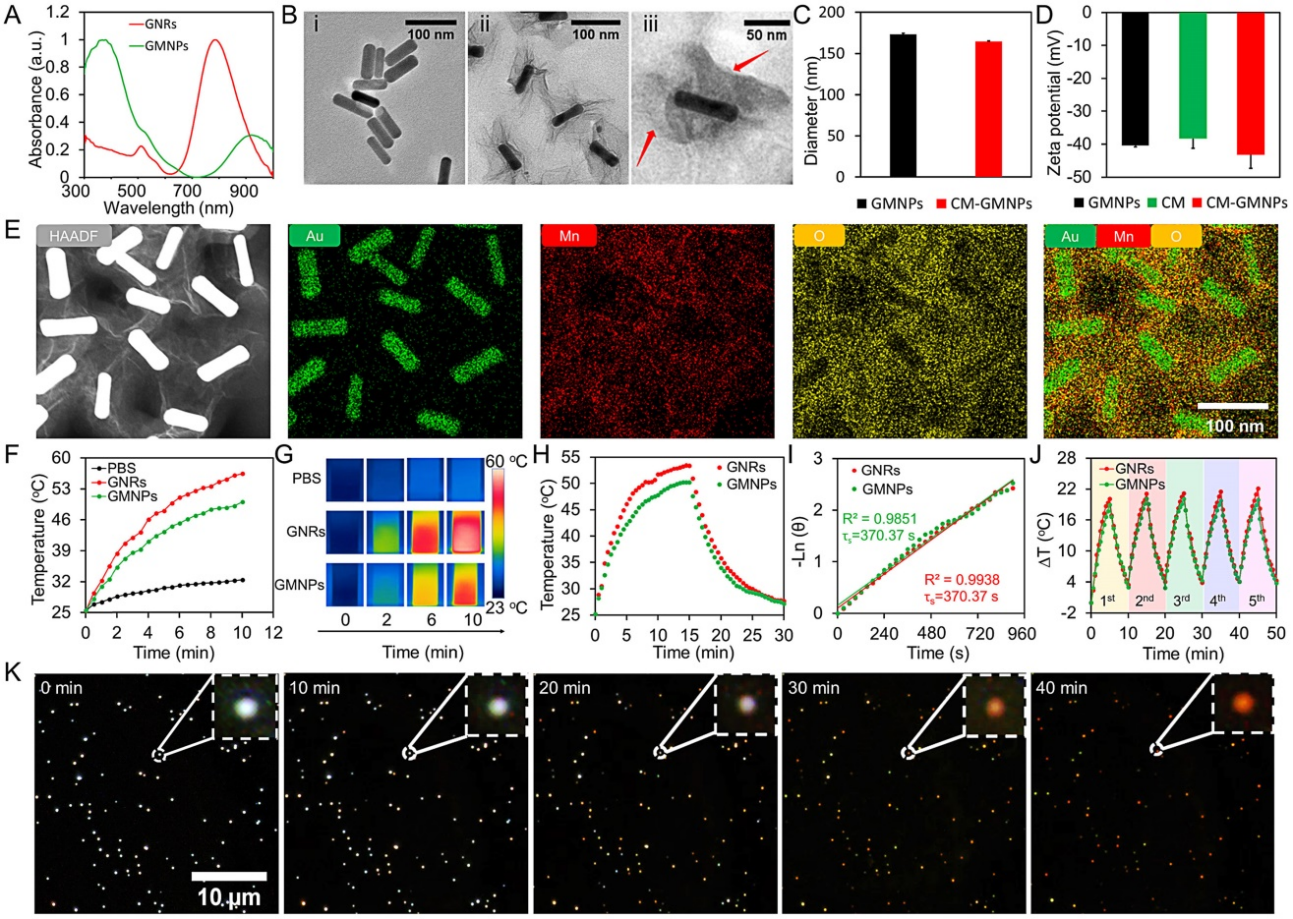
(A) UV-vis absorption spectra of GNRs and GMNPs. (B) TEM images of GNRs (i), GMNPs (ii) and CM-GMNPs (iii). (C) Hydrodynamic size of GMNPs and CM-GMNPs. (D) Zeta potential of GMNPs, cell membrane fragments (CM) and CM-GMNPs. (E) From left to right are the high resolution TEM image of GMNPs and elemental mapping analysis of GMNPs for Au, Mn, O and the merged image. (F) Temperature elevation curves of PBS (black), GNRs (red) and GMNPs (green) upon 808 nm laser irradiation (1.5 W cm^-2^). (G) The corresponding infrared thermal images of PBS, GNRs, and GMNPs. (H) Photothermal responses of GNRs (red) and GMNPs (green) with concentration of 35 pM under NIR laser irradiation for 900 s. (I) Linear time *versus* -ln θ obtained from the cooling period. (J) Photostability of GNRs (red) and GMNPs (green) under 808 nm laser irradiation (1.5 W cm^-2^). (K) Dark-field optical microscopic images of GMNPs in the presence of 0.05 μM GSH as a function of time.

In this work, the 2D supraparticle GMNPs were synthesized through an *in situ* self-assembly method 32. In brief, GNRs (71×23 nm) were firstly obtained according to a seeded-mediated growth method 33, 34. To coat MnO_2_ nanosheet, GNRs were treated with poly(sodium-p-styrenesulfonate) (PSS) solution overnight to alter the surface potential. Then, sodium citrate (5 mM) and KMnO_4_ (10 mM) were sequentially introduced into the purified GNRs solution. The MnO_2_ layer was finally deposited onto the GNR surface by refluxing the mixture at 35 °C for 24 h. As shown in Figure 1A, an absorption peak at ~380 nm appears in the ultraviolet-visible (UV-vis) absorption spectrum (the green line), which is ascribed to the formation of MnO_2_ nanosheets. At the same time, the characteristic local surface plasmon resonance (LSPR) peak of GNRs shifts from ~790 to ~930 nm, further confirming the generation of MnO_2_ layer on the GNRs surface. From the transmission electron microscope (TEM) images in Figure 1B (i and ii), distinct 2D MnO_2_ nanosheets wrap around the outside of GNRs with desirable uniformity, mono-dispensability and well-defined core-shell structure. According to the dynamic light scattering (DLS) results, the hydrodynamic diameter of GMNPs is 173.4 ± 1.1 nm and zeta potential is -40.3 ± 0.4 mV (Figure 1C and 1D). Elemental mapping analysis from the high resolution TEM also shows that a thin layer of MnO_2_ nanosheet is coating on the surface of GNR (Figure 1E). Furthermore, X-ray photoelectron spectroscopy (XPS) analysis was used to measure the elemental composition of GNRs and GMNPs. From the XPS spectra (Figure S1), GNRs are mainly composed of Au atoms. However, when the surface of GNRs was coated with MnO_2_ nanosheets, a characteristic peak of Mn is observed. In the high-resolution XPS spectra, the binding energies at 639.5 and 650.9 eV from Mn 2p band imply the presence of Mn. Additionally, the peak at 527.8 eV (Mn-O-Mn) in O 1s spectrum also confirms the successful coating of MnO_2_.

In order to investigate whether the presence of MnO_2_ layer affects the photothermal performance of GNRs, the photothermal conversion effect of GNRs and GMNPs in the NIR region was explored. On this basis, GNRs and GMNPs with concentration of 35 pM were exposed to 808 nm NIR laser with the laser power of 1.5 W cm^-2^ for 600 s. The temperature of the solutions increased to 56.4 and 50.0 °C respectively for these two samples (Figure 1F and 1G). This is noticeably different from the result in phosphate buffer saline (PBS) solution. To further investigate the photothermal conversion efficiency, different concentrations of these two samples under the same laser irradiation condition were explored. The heating curves show concentration-dependent photothermal effect (Figure S2). The photothermal transduction efficiencies (η) of GNRs and GMNPs are calculated to be 39.2% and 46.9%, respectively (Figure 1H and 1I). It is worth to note that these results are determined at the wavelength of 808 nm. The absorption coefficient of GMNPs is lower than that of GNRs at this wavelength, which results in relatively higher photothermal transduction efficient for GMNPs at this wavelength according to the equation as shown in the supporting information. Importantly, the photothermal effect of GNRs and GMNPs remains good after five cycles of heating and cooling (Figure 1J). This is further confirmed by the spectroscopic measurements, where the morphology of UV-vis spectra of GNRs and GMNPs didn't change after laser irradiation (Figure S3), revealing the excellent photostability of these samples.

According to earlier explorations, MnO_2_-based nanostructures have sensitive responsiveness regarding endogenous GSH or acidic H_2_O_2_ 35. To verify this point, we recorded *in situ* dark-field optical microscopic images of GMNPs in the presence of either 0.05 μM GSH or H_2_O_2_ (pH=5.5) as a function of time, respectively 36, 37. As shown in Figure 1K and S4, the color of GMNPs gradually changes from bright white to dark, and finally to red (the color of GNRs in the dark-field microscopic image, Figure S5) after 40 min. Elemental mapping result of GMNPs after etching process also confirms the complete etching of MnO_2_ nanosheets on the rod surface (Figure S6).

### Preparation, characterization and homotypic targeting validation of CM-GMNPs

Specific tumor targeting capability and excellent colloidal stability are two essential factors to evaluate the performance of the nanodrug for cancer therapy. Interestingly, strong affinity has been found between circulation tumor cells, resulting the aggregation and formation of solid tumor 28, 38. This homotypic accumulation is highly associated with the recognition and interaction of various molecules and proteins on the cell membrane 28. Considering the potential tumor targeting capability of cancer cell membrane, we coated GMNPs with cell membranes (by using 4T1 cell as a model) to endow the nanoparticles with improved biological characteristics in complex physiological environment, especially the colloidal stability and tumor specificity 38, 39.

To coat cancer cell membrane onto the surface of GMNPs, cell membrane fragments were first extracted from 4T1-breast-cancer cells with a membrane protein extraction kit and centrifugation 28. The CM-GMNPs were then obtained by extruding the mixture of cracked cell membrane and GMNPs solution through a filter with pore size of 400 nm for at least 5 times. The TEM images reveal the morphologic features of formed nanoparticles. As shown in Figure 1B (iii), CM-GMNPs display a typical core-shell structure with cell membrane as the shell (red arrows). It is worth noting that the MnO_2_ layer does not spread out around the GNR as that from GMNP. A relatively compact structure was observed, indicating that the MnO_2_ nanosheet is compressed during the extrusion process. As confirmed by the DLS characterization in Figure 1C, the average hydrodynamic diameter of CM-GMNPs is 164.5 ± 0.7 nm. Apparently, the size is a little bit smaller than that of GMNPs, which is in good agreement with the TEM results. Besides, zeta-potential of CM-GMNPs is determined to be -43.2 ± 4.0 mV, which also changes slightly (Figure 1D).

To further validate the cell membrane coating process, additional spectroscopic measurements were performed. As shown in the Fourier transform infrared (FT-IR) spectra in Figure S7, the peaks at 1061, 1236, and 1739 cm^-1^ in the spectrum of CM-GMNPs correspond to C-O-C stretching vibration, PO_2_^-^ stretching vibration and C=O stretching vibration respectively, which are the characteristic groups from lipid membrane. In the Raman spectroscopic analysis (Figure S8), distinct peaks at 635 and 2935 cm^-1^ are assigned to the Mn-O stretching vibration and the superposition of C-H symmetric and asymmetric stretching vibrations of cell membrane respectively. These observations give clear evidences on the successful coating of desired MnO_2_ nanosheet and cell membrane. More importantly, direct colocalization analysis at the single-particle level with optical microscopy was performed to verfiy the observations as noted above. In this experiment, the cell membrane was co-stained with fluorescent dye DiO. As shown in Figure S9, the fluorescent spots from cell membrane (green channel) perfectly overlap with the scattering light from each GMNPs (red channel) in the dark-field optical microsopic image. This single-particle imaging result gives a direct and solid proof on the successful coating of GMNPs with the cell membrane.

The colloidal stability of CM-GMNPs was explored with dark-field optical microscopy at the single-particle level. As shown in Figure S10, without membrane coating, GMNPs show good monodispersity in H_2_O, while aggregate into large agglomerates in PBS solution and dulbecco's modified eagle medium (DMEM). After being coated with cell membrane, the particles exhibit comparable monodispersity in H_2_O, PBS solution, and DMEM, indicating the good colloidal stability of CM-GMNPs. Statiscally analyzed scattering intensity distributions from the particles in these three media further validate the good colloidal stability of CM-GMNPs, enabling their usage in biological medium. In a word, these results give clear evidence on the successful coating of cell membrane on the surface of GMNPs.

**Figure 2:**
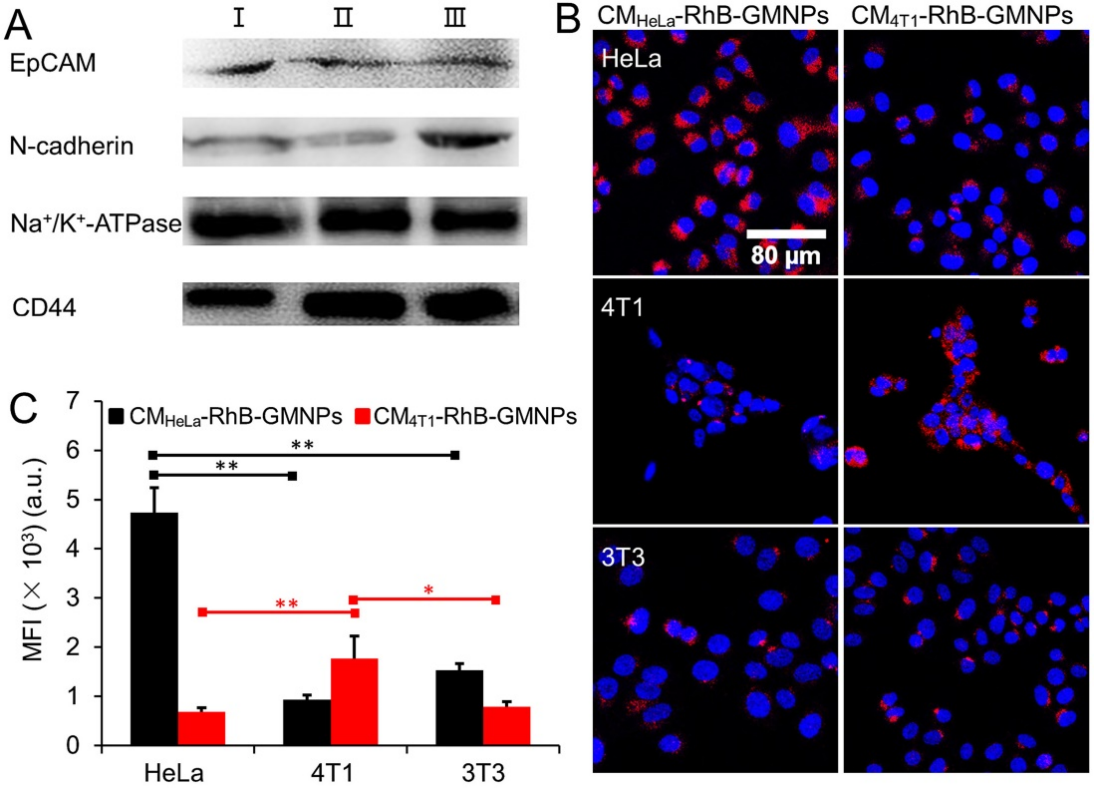
(A) Western blotting analysis of cancer cell lysate (Ⅰ), cell membrane fragments (Ⅱ) and CM-GMNPs (Ⅲ) regarding four cell adhesion molecules associated-antibodies. (B) LCSM images and (C) corresponding mean intensity of red fluorescence from HeLa, 4T1 and 3T3 cells which were co-incubated with CM_HeLa_-RhB-GMNPs and CM_4T1_-RhB-GMNPs, respectively (p*<0.05; p**<0.01).

Decoration of cancer cell membrane protein to the nanoparticle shell has been shown to improve the biological property of the nanoparticle, especially the homotypic recognition ability 29, 39. In this regard, western blotting experiments were next carried out to confirm the existence of membrane proteins by using four cell membrane adherence molecular related antibodies, including EpCAM, N-cadherin, Na^+^/K^+^-ATPase and CD44 28, 40, 41. As depicted in Figure 2A, 4T1 cells lysate (Ⅰ), cell membrane fragments (Ⅱ) as well as CM-GMNPs (Ⅲ) exhibit similar protein bands, implying that the membrane proteins are successfully transferred to CM-GMNPs.

Considering that the MnO_2_ nanosheets on GMNPs possess flexible ultra-thin structure and large surface area, distinctive negative charge as well as degradable property in cellular environment, we expect that CM-GMNPs can be used as an intelligent carrier for stimuli responsive drug delivery. In order to verify this point, a positively charged dye, Rhodamine B (RhB), was absorbed on the surface of GMNPs as a model molecule. As shown in the UV-vis absorption and fluorescence spectra in Figure S11A and S11B, characteristic peaks of RhB are found in RhB-GMNPs and CM-RhB-GMNPs. Noticeably, the fluorescence intensity of adsorbed RhB from the resulted solution is obviously lower than that from free RhB with the same concentration in the solution as shown in Figure S11C. Given the MnO_2_ layer is etched by GSH, the fluorescence intensity is recovered to the normal level immediately. Additionally, to characterize the dye loading process in a more intuitive way *in vitro*, cell membrane fragments were labeled with fluorescent dye DiO (green) and encapsulatd with RhB-GMNPs. After 1 h of co-incubation, 4T1 cells were imaged with laser confocal scanning microscope (LCSM). Obvious overlapping between RhB and DiO is observed in the fluorescence microscopic image (Figure S12). These results confirm the successful loading, encapsulation and releasing of RhB, indicating that CM-GMNPs can be served as stimuli-responsive drug carriers as expected.

Encouraged by the western blotting results, we next investigated the homotypic cancer cell recognition ability of CM-GMNPs. CM_HeLa_-RhB-GMNPs and CM_4T1_-RhB-GMNPs were utilized to incubate with HeLa cells, 4T1 cells and 3T3 cells for 1 h. From the LCSM images in Figure 2B, as expected, the fluorescent intensity of HeLa cells co-cultured with CM_HeLa_-RhB-GMNPs and 4T1 cells co-cultured with CM_4T1_-RhB-GMNPs are much higher than that from other cell samples. The statistical results further reveal that the intracellular fluorescence intensity of the sample treated with the same membrane coated GMNPs is 2-5 times higher than that from other samples (Figure 2C). These results illustrate that the introduction of cell membrane endows the nanoparticle with greatly improved targeting ability toward homologous cells, which can be ascribed to the successful transferring of membrane proteins and other recognition associated substance.

### Cytotoxicity, *in vitro* drug-releasing, and antiproliferative effects of CM-DOX-GMNPs

**Figure 3:**
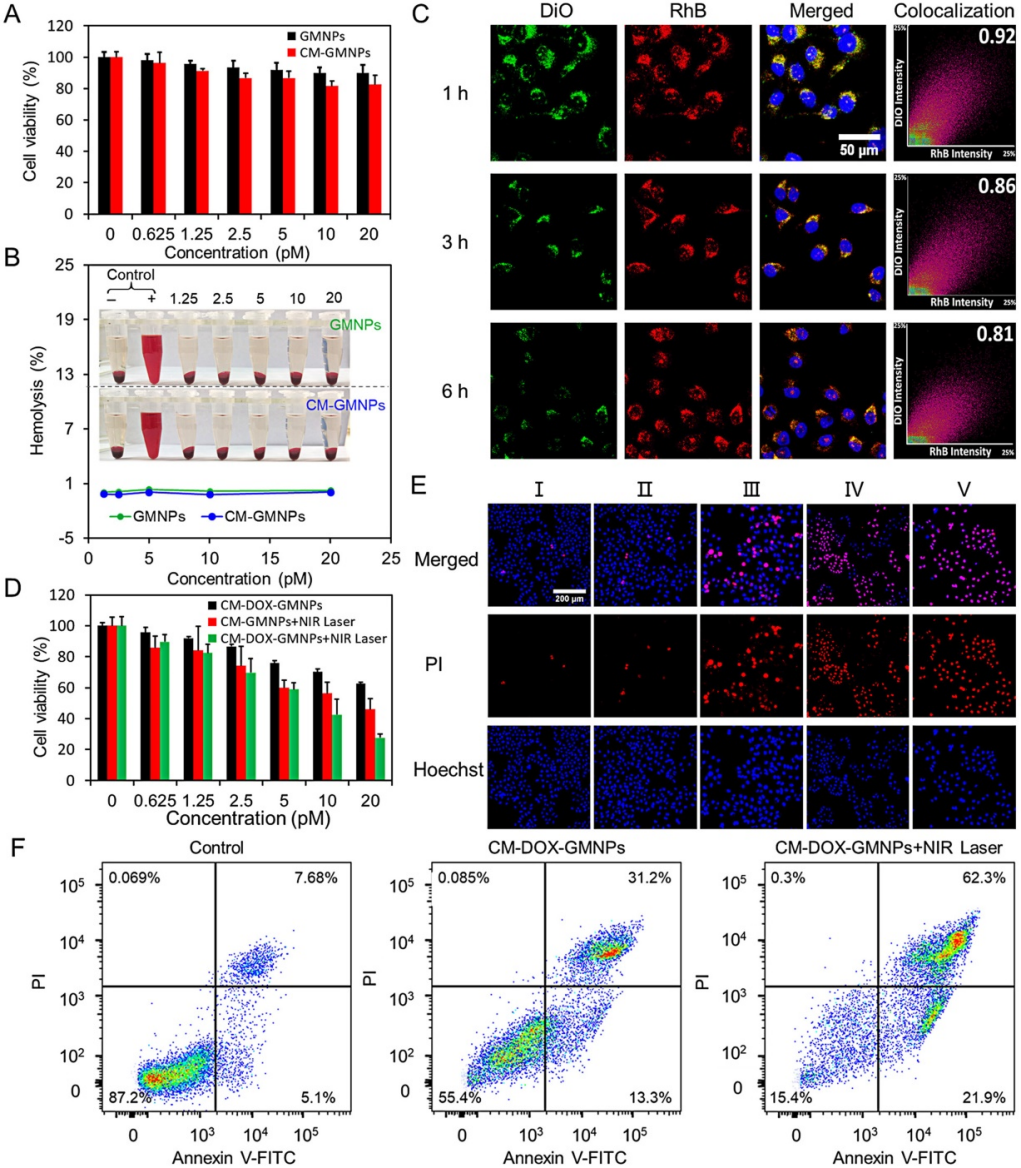
(A) The cytotoxicity assay of GMNPs and CM-GMNPs at various concentrations. (B) Hemolysis of GMNPs (green) and CM-GMNPs (blue) to red blood cells. (C) LCSM images and corresponding colocalization profiles of 4T1 cells stained with Hoechst and co-cultured with CM-RhB-GMNPs for 1, 3 and 6 h. (D) The cell viability assay of 4T1 cells treated with CM-DOX-GMNPs, CM-GMNPs+NIR laser and CM-DOX-GMNPs+NIR laser. (E) LCSM images of 4T1 cells treated with cell culture medium (Ⅰ), NIR laser (Ⅱ), CM-DOX-GMNPs (Ⅲ), CM-GMNPs+NIR laser irradiation (Ⅳ), and CM-DOX-GMNPs+NIR laser (V). All cells were stained with Hoechst (blue) and the dead cells were stained with PI (red). (F) Flow cytometry analysis on cells treated with CM-DOX-GMNPs with and without NIR irradiation. From left to right are the control group, the cells treated with CM-DOX-GMNPs only, and the cells treated with CM-DOX-GMNPs under 808 nm laser irradiation.

Biocompatibility is an important parameter to evaluate the performance of the nanodrug. The cytotoxicity of GMNPs and CM-GMNPs was then performed based on the 3-(4,5-dimethylthiazol-2-yl)-2,5-diphenyltetrazolium bromide (MTT) assay. As shown in Figure 3A, all cells display a survival rate more than 80% after being incubated with GMNPs and CM-GMNPs for 24 h with the concentration <20 pM, suggesting satisfied biocompatibility of the nanoparticles. Compared with GMNPs, the cytotoxicity of CM-GMNPs is slightly higher at each concentration. It is largely ascribed to the cell membrane associated-specific recognition and binding between CM-GMNPs and homologous cancer cells. As verified by dark-field optical microscopic images in Figure S13, the cellular internalization amount of CM-GMNPs is much higher than that of membrane-free GMNPs. Furthermore, hemolysis experiment was executed to evaluate the biocompatibility of the nanostructures. As shown in Figure 3B and S14, the hemolysis of GMNPs and CM-GMNPs at the maximum experimental concentration (20 pM) is <0.3% and <0.1% respectively, suggesting the good biocompatibility of these nanostructures.

Effective drug-releasing is one of the essential indicators reflecting the efficacy of drug delivery system. On this account, DiO labeled CM-RhB-GMNPs were utilized to track the cargo-releasing process by co-incubating with 4T1 cells for 1, 3 and 6 h. As shown in Figure 3C, the green fluorescence from cell membrane shells and the red fluorescence from the cores co-localizes well with the Mander's overlapping coefficient of 0.92. However, as the co-incubation time increasing, the intensity from these two channels decreases gradually, especially in the green channel. Meanwhile, the degree of co-localization also reduces to 0.81 after 6 h of co-incubation. From these results, we can infer that the CM-RhB-GMNPs retain their integrity and stability before and after cellular internalization, which avoids undesired nanoparticles dissociation and drug leakage. However, within the intracellular environment, the shell of cell membrane is gradually cracked, leading to the reduction of MnO_2_ nanosheets and the subsequent releasing of drug molecules.

Encouraged by the favorable outcomes regarding photothermal conversion effect, drug-releasing property and biocompatibility, the antiproliferative effects of the nanoparticles were first investigated *in vitro*. Anticarcinogen DOX was loaded onto the surface of GMNPs. UV-vis absorption spectra in Figure S15 show the characteristic peak of DOX before and after cell membrane coating, demonstrating the successful adsorption and encapsulation of DOX. Benefited from the large surface area and strong negative potential of MnO_2_ nanosheets, GMNPs provide a drug loading efficiency of 12.1 ± 0.3%, and a releasing ratio of 44.8 ± 3.0% according to the quantitative spectroscopic measurements. The therapeutic efficacy was examined based on cell viability outcomes by treating 4T1 cells with CM-DOX-GMNPs, CM-GMNPs+NIR laser, CM-DOX-GMNPs+NIR laser. As described in Figure 3D, only about 38% and 55% of cells in the first two groups died, while more than 70% of cells died in the last group.

LCSM imaging experiments and corresponding fluorescence intensity statistics within cells were further performed to study the antiproliferative effect of this nanodrug in a more intuitive way. Here, the dead cells were stained with PI (red). As shown in Figure 3E and Figure S16, compared with the control group where the cells are only co-incubated with culture medium, no evident death is detected in the group treated with 808 nm NIR laser, indicating that the laser has negligible effect on the cell viability. Furthermore, dead cells in the CM-DOX-GMNPs group are distinctly less than that in the CM-GMNPs+NIR laser group, implying PTT possesses a synergistic effect on the inhibition performance towards cancer cells proliferation compared with chemotherapy alone. Besides, almost all cells incubated with CM-DOX-GMNPs are destroyed to death upon irradiation with 808 nm laser, which is also consistent with the MTT assay as observed above. Consequently, CM-DOX-GMNPs with NIR laser can bring the best antiproliferative outcomes. These observations are in good agreement with the flow cytometry results as shown in Figure 3F.

### *In vivo* MRI, PTI and antiproliferative efficacy

**Figure 4:**
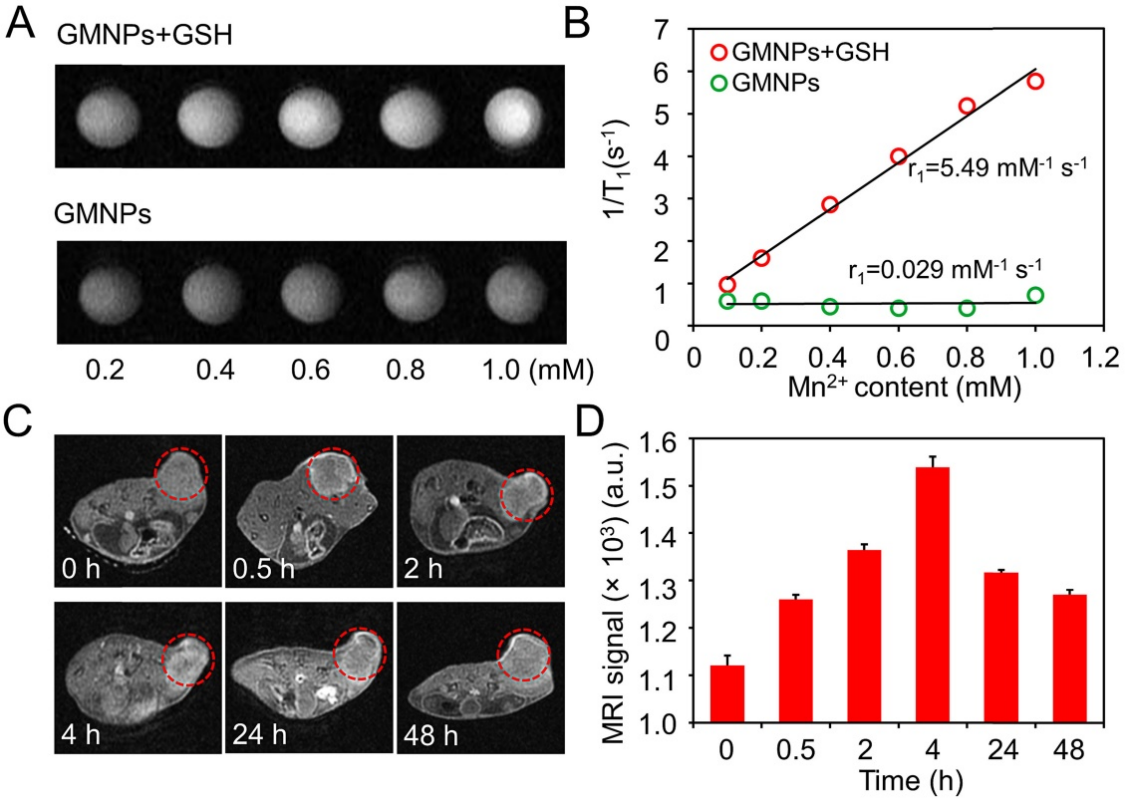
(A) T_1_-MR phantom images and (B) corresponding longitudinal relaxivities of GMNPs suspensions at different Mn concentrations in the presence and absence of GSH. (C) *In vivo* MR images at different time (the tumor areas are indicated by red circles). (D) Corresponding MRI signal value of tumor region at different time after intravenous injection.

Since the MnO_2_ layer on the GNRs surface can be effectively reduced to Mn^2+^, this nanostructure has the potential in serving as contrast agents for enhanced T_1_-weighted MR imaging 30, 42. Therefore, the relaxation property of CM-GMNPs was investigated to evaluate the effectiveness for MRI. Figure 4A shows the MR images of the GMNPs in the presence and absence of GSH. Obviously, the MR signal of GMNPs with GSH is much higher than that of the nanoparticles in neutral aqueous solution. Meanwhile, the signal intensity increases gradually with the increment of Mn content. The longitudinal relaxivity r_1_ of GMNPs is obtained *via* the linear fitting of 1/T_1_ and Mn^2+^ concentration, which exhibits huge enhancement effect with a value of 5.49 mM^-1^ s^-1^ (Figure 4B). In brief, the GMNPs display favorable MRI ability with distinct Mn^2+^ concentration-dependent sensitivity as well as high relaxivity value.

The *in vivo* T_1_-weighted MRI behavior of CM-GMNPs was characterized by intravenously injecting the nanoparticles into 4T1 breast tumor-bearing mice. From the MRI images and corresponding signals in Figure 4C and 4D, no evident difference is noted between tumor tissue and normal tissue. However, remarkable signal response is observed in the tumor area after 0.5 h injection, and it reaches maximum at 4 h with the value of 1539. In addition, the tumor area still displays evident brightness even after 48 h. These observations demonstrate that CM-GMNPs can be effectively enriched in tumor area in a short time and possess good imaging capability. Meanwhile, the property of long retention time at the target location may be instructive for long-time tracking.

**Figure 5:**
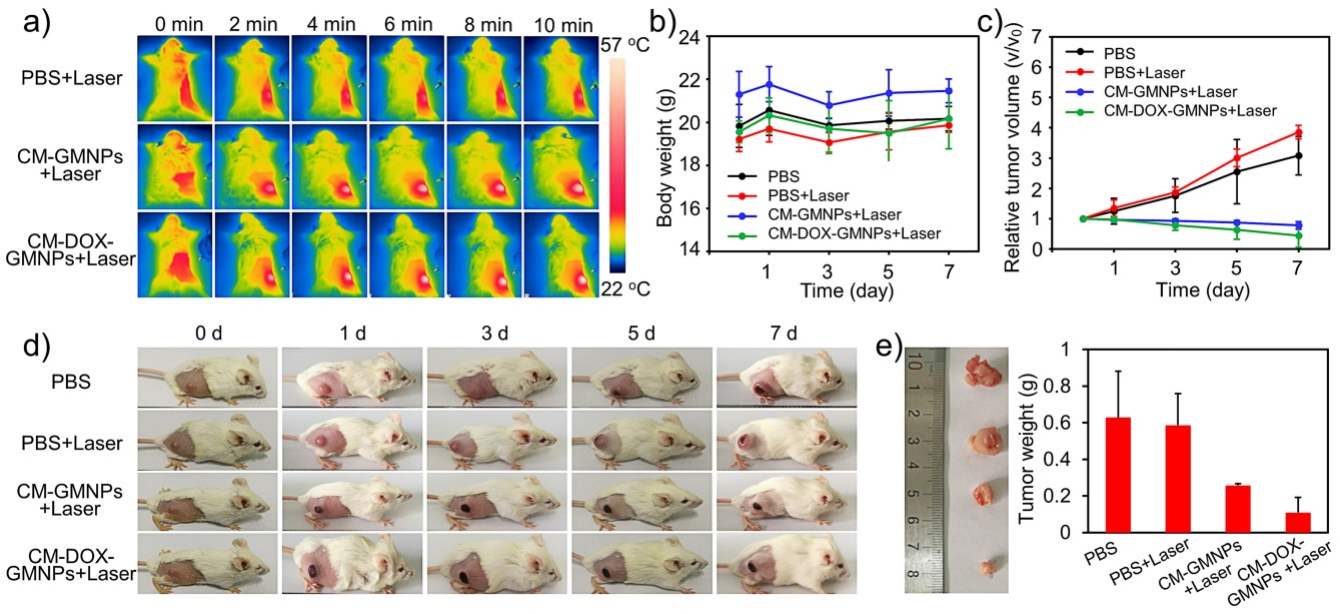
(A) Infrared thermographic images of 4T1 breast tumor-bearing mice treated with PBS, CM-GMNPs and CM-DOX-GMNPs in the presence of NIR laser at different irradiation time. (B) Body weight changes and (C) relative tumor volume of 4T1 breast tumor-bearing mice treated with PBS, PBS+NIR laser, CM-GMNPs+NIR laser and CM-DOX-GMNPS+NIR laser within 7 days. (D) Photographs of the mice in different groups. (E) The photograph and corresponding weight of tumors which were collected on the 7th day.

The PTI and antiproliferative efficacy *in vivo* was tested by using 4T1 breast tumor-bearing mice. The mice were first divided into three groups and were intravenously injected with 200 μL of PBS solution, CM-GMNPs and CM-DOX-GMNPs (5 mg·mL^-1^), respectively. After 4 h, all mice were exposed to 808 nm laser with a power density of 1 W·cm^-2^ for 10 min. The infrared thermographic images are shown in Figure 5A. The temperature of tumor areas in the treated mice increases apparently within 2 min, and finally reaches to almost 57 °C, which is high enough to induce the cell death. In contrast, the temperature of the control group shows only a slight enhancement with the same exposure time. These results demonstrate the excellent heat generation ability from CM-GMNPs and CM-DOX-GMNPs, which can be used for PTI.

To further evaluate the antitumor efficacy, the mice treated with PBS, PBS+NIR laser, CM-GMNPs+NIR laser and CM-DOX-GMNPs+NIR laser were monitored for 7 days. As show in Figure 5B, the body weight changes of all groups fluctuate within a small range, suggesting negligible toxicity from NIR laser, CM-GMNPs and CM-DOX-GMNPs. However, changes in tumor regions from these four groups are evidently different from each other. In detail, the relative tumor volume of the mice in the control group and laser illumination group increases by more than 200%, while obvious decrease is observed from the mice treated with CM-GMNPs and CM-DOX-GMNPs in the presence of NIR 808 nm laser illumination (Figure 5C and 5D). In particular, the CM-DOX-GMNPs+NIR laser group displayed 55% reduction in relative tumor volume, which is noticeably better than 22% reduction in the CM-GMNPs+NIR laser group. Finally, we also harvested the tumors of different mice. As depicted in Figure 5E, the tumor size and weight of the mice treated with CM-DOX-GMNPs and 808 nm laser are much smaller than that of other three groups. From the above results, we inferred that the CM-DOX-GMNPs possess a strong inhibition and therapeutic effect on the tumor in the presence of laser, which is largely resulted from the combination of chemotherapy and PTT.

### Biodistribution and toxicity studies

Biological toxicity of the nanocargo is one of the crucial factors which will influence the biomedical application of nanomaterials. Hence, we further evaluate the systematic toxicity of CM-GMNPs *in vivo*. The mice were first divided into four groups randomly. Then three of the groups were intravenously injected with 200 μL of CM-GMNPs solution (5 mg·mL^-1^) and sacrificed on the 1st, 7th and 14th days. The last group without treatment was also sacrificed on the 14th days and was served as a control group.

**Figure 6:**
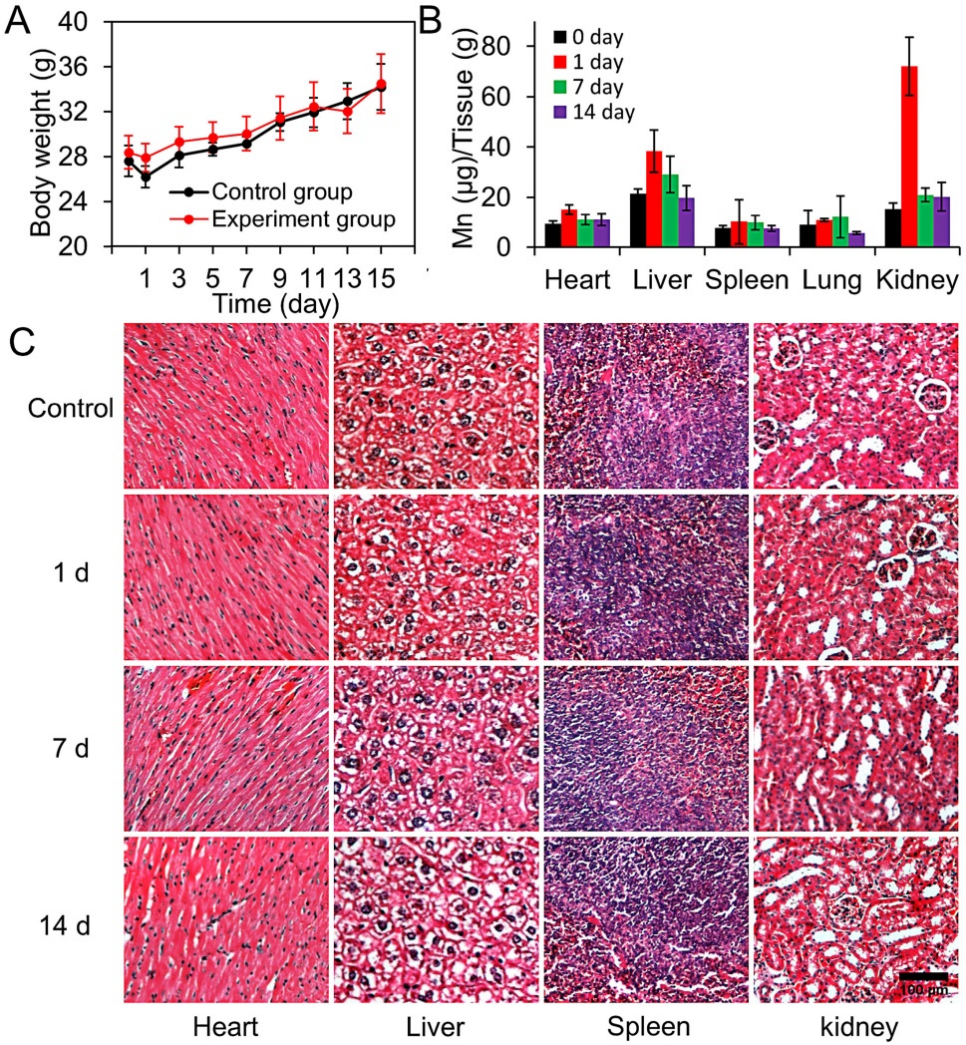
(A) Body weights of the mice in the control and experimental groups which were intravenously injected with 200 μL of CM-GMNPs (5 mg mL^-1^) in 15 days. (B) The biodistribution measurements of Mn^2+^ content in various organs of mice treated with CM-GMNPs for 0, 1, 7 and 14 days. (C) H&E staining images of hearts, livers, spleens and kidneys of mice treated with CM-GMNPs for 0 (control), 1, 7 and 14 days.

The body weights changes of mice in the control group and experimental group are shown in Figure 6A. Apparently, the body weight of mice in these two groups present similar variation tendency over a span of 14 days, indicating that the nanoparticles have no clear interference on the growth of the mice. Besides, we measured the Mn^2+^ content in major organs using inductively coupled plasma mass spectrometry (ICP-MS) to explore the biodistribution of nanoparticles (Figure 6B). As expected, the amount of CM-GMNPs in the liver and kidney is much higher than that in other organs, which is owing to the uptake of CM-GMNPs in macrophages. Meanwhile, we are informed that the Mn^2+^ content gradually decreases over time and finally recovers to a relative low level, demonstrating the CM-GMNPs can be effectively eliminated from organism. The histological examinations were performed to determine the histological toxicity of CM-GMNPs. As shown in Figure 6C, no distinct organ lesions, inflammation, or abnormalities are observed in all the tested organs from hematoxylin and eosin (H&E)-stained slices, further certifying that the CM-GMNPs possess superior biocompatibility for *in vivo* theranostic applications in the future.

## Methods

### Preparation of GNRs, GMNPs and CM-GMNPs

GNRs were synthesized according to a modified seed mediated growth method 33, 34. Typically, the seed solution was first prepared by adding 0.25 mL of HAuCl_4_ solution (0.01 M) into 10 mL of CTAB (0.1 M). Then, 0.6 mL of freshly prepared ice-cold NaBH_4_ solution (0.01 M) was injected into the mixture quickly. The seed solution was obtained after maintaining the as-prepared solution at 30 °C for 2 h. For the growth of GNRs, 7.5 mL of HAuCl_4_ solution (0.01 M), 1.2 mL of AgNO_3_ solution (0.01 M), 3 mL of HCl (0.1 M) and 1.2 mL of ascorbic acid (0.1 M) were successively added into 150 mL of CTAB (0.1 M) solution and mixed sufficiently. Afterwards, the growth solution was mixed with 0.21 mL of as-prepared seed solution and kept at 30 °C overnight. Finally, the products were collected by centrifugation and washed with deionized water.

GMNPs were obtained by a modified *in situ* self-assembly method 32. In brief, the GNRs were incubated with Poly(sodium-p-styrenesulfonate) (PSS) solution overnight. After being centrifugated, the precipitate was dispersed in sodium citrate (5 mM) solution, and then KMnO_4_ solution (10 mM) was added under stirring. The mixture was finally heated to 35 °C and refluxed for 24 h. GMNPs were obtained by centrifugation.

In order to prepare CM-GMNPs, the cell membrane fragments were first obtained through a membrane protein extraction kit 28. In detail, cells were incubated in cell culture dishes to reach an appropriate density. After being centrifuged at 600 ×g for 5 min at 4 °C and washed with PBS solution for two times, the harvested cells were suspended in the hypotonic lysing buffer containing protease inhibitor, and were incubated at 4 °C for 15 min. The cells in the above solution were then broken repeatedly until 80% of them lost complete structure under microscope. Subsequently, the homogenized solution was centrifuged at 700 ×g for 10 min at 4 °C to remove cell nucleus. The supernatant was further centrifuged at 14,000 ×g for 30 min to obtain cell membrane fragments. The CM-GMNPs were obtained by co-extruding the cell membrane fragments and nanoparticles solution through a 400 nm polycarbonate membrane for at least 5 times. CM-DOX-GMNPs were prepared by the same procedures but GMNPs were suspended in PEG solution (1 μg mL^-1^) and were co-incubated with DOX solution (1 mg mL^-1^) for 3 h before use.

### Dark-field microscopic imaging

The *in situ* dark-field microscopic characterizations of nanoparticles were performed using a dark-field optical microscope (Ti-U, Nikon, Japan) equipped with a 40× objective (NA=0.75) 36, 37. In detail, the nanoparticles were first fixed on the glass-substrate to form reaction channels. Then GSH solution or H_2_O_2_ solution (0.05 μM, pH=5.5) were injected into the channels. The images in different time points were captured by a true-color complementary metal oxide semiconductor camera (sCMOS, Digiretina 16, Tuscen photonics Co., Ltd., China). The stability of GMNPs and CM-GMNPs in different media were measured by the same method and apparatus. All data were analyzed by the image processing software, Image J (http://rsb.info.nih.gov/ij/).

### *In vivo* MRI imaging

*In vivo* experiments were carried out in the 4T1 breast tumor model. First, the model was established by subcutaneous plantation of 4T1 cells on the back of the Balb/c mice (Beijing HFK Bioscience Co. Ltd., China). After the tumor size reached to 6-8 mm, the mice were anesthetized with chloral hydrate solution (4 wt.%), followed by intravenously injecting with 200 μL GMNPs solution (5 mg mL^-1^). MRI scans were performed at different time intervals on a 3.0 T MRI system (GESigna Excite) that was equipped with a special animal coil.

### *In vivo* PTI and antiproliferative effect study

4T1 breast tumor models were first established on Balb/c mice. After the tumor size reached to 6-8 mm, the mice were randomly divided into four groups with 3 mice in each group. Then the mice of the first two groups were intravenously injected with 200 μL of PBS solution (10 mM, pH=7.4). Group three were intravenously injected with 200 μL of CM-GMNPs (5 mg mL^-1^) while the last group were intravenously injected with 200 μL of CM-DOX-GMNPs (5 mg mL^-1^). After 4 h, except the first group, all mice were exposed to 808 nm laser (1 W cm^-2^) for 10 min, and the photothermal images were collected by an infrared camera every 2 min.

For the antiproliferative effect study, the body weight and tumor volume of all mice were strictly recorded every other day. After 7 days of treatment, all mice were sacrificed. Their tumors were collected for taking photo and weigh measurement.

### Biodistribution and toxicity studies

The mice were randomly divided into four groups with four mice in each group. Specifically, the first group was set as control group while another three were intravenously injected with 200 μL of CM-GMNPs (5 mg mL^-1^). Two of experimental groups were sacrificed at the 1st and the 7th day post-injection. After each day of weighing, the mice in the last group were also sacrificed at 14th day. For the biodistribution study and histological analysis, major organs (heart, liver, spleen, lungs, and kidneys) were collected. The Mn levels were measured by ICP-MS (Thermo Scientific, Madison, USA), and the histological analysis was conducted using paraffin slices and hematoxylin and eosin (H&E) staining.

## Conclusion

In summary, we demonstrate an intelligent 2D supraparticle CM-DOX-GMNPs which integrates a DOX-GMNP core and cancer cell membrane shell for tumor targeted MRI/PTI dual-modal imaging and chemo/photothermal therapy. The CM-DOX-GMNPs display specific tumor targeting ability and excellent colloidal stability in cellular uptake process. Within the cell, MnO_2_ nanosheets on GNRs surface are successfully etched to Mn^2+^ by endogenous GSH or acidic H_2_O_2_, leading to the stimuli-controlled release of DOX for tumor specific chemotherapy. The etched Mn^2+^ within the tumor area is further used as MRI agent for *in vivo* tumor imaging. Moreover, due to the efficient photothermal conversion effect from GNRs, under NIR laser illumination, photothermal therapy is thus activated for the synergistic cancer treatment with DOX in 4T1 breast tumor-bearing mice. Enhanced tumor accumulation, and greatly improved tumor inhibition effects are confirmed *in vivo*. The nanostructure described here offers a robust approach for the diagnosis and treatment of cancer, which displays the potential to circumvent the ongoing challenges associated with singlet cancer therapeutic strategies such as PTT or chemotherapy.

## Supplementary Material

Supplementary figures and tables.

## Abbreviations

MRI: magnetic resonance imaging
PTI: photothermal imaging
GNR: gold nanorod
DOX: doxorubicin
GSH: glutathione
NIR: near-infrared
PTT: photothermal therapy
PTAs: photothermal transduction agents
TME: tumor microenvironment
MRI: magnetic resonance imaging
LSPR: local surface plasmon resonance
TEM: transmission electron microscope
XPS: X-ray photoelectron spectroscopy
DLS: dynamic light scattering
FT-IR: Fourier transform infrared
UV-vis: ultraviolet-visible
DMEM: dulbecco's modified eagle medium
PBS: phosphate buffer saline
RhB: Rhodamine B
LCSM: laser confocal scanning microscope
MTT: 3-(4,5-dimethylthiazol-2-yl)-2,5-diphenyltetrazolium bromide
ICP-MS: inductively coupled plasma mass spectrometry
H&E: hematoxylin and eosin
