# Supplementary material for: Cell membrane coated smart two-dimensional supraparticle for in vivo homotypic cancer targeting and enhanced combinational theranostics
Source: Nanotheranostics. 2021 Feb 14;5(3):275–87. doi: 10.7150/ntno.57657 (PMC7914337; doi:10.7150/ntno.57657)
Supplement: Supplementary file 1 — Supplementary figures and tables. [file ntnov05p0275s1.pdf]

Supporting Information

Cell membrane coated smart two-dimensional supraparticle for *in vivo* homotypic cancer targeting and enhanced combinational theranostics

Di Zhang[1,2#], Zhongju Ye[1,2#], Hua Liu[2], Xin Wang[2], Jianhao Hua[2], Yunyun Ling[3], Lin Wei[4], Yunsheng Xia[3✉], Shaokai Sun[5✉], and Lehui Xiao[2✉]

1. College of Chemistry, Zhengzhou University, Zhengzhou, 450001, China

2. State Key Laboratory of Medicinal Chemical Biology, Tianjin Key Laboratory of Biosensing and Molecular Recognition, College of Chemistry, Nankai University, Tianjin, 300071, China

3. Key Laboratory of Functional Molecular Solids, Ministry of education, College of Chemistry and Materials Science, Anhui Normal University, Wuhu, 241000, China

4. College of Chemistry and Chemical Engineering, Hunan Normal University, Changsha, 410081, China

5. School of Medical Imaging, Tianjin Medical University, Tianjin, 300071, China

#These authors contributed equally to this work.

🖂 Corresponding author

Email: xiayuns@mail.ahnu.edu.cn; shaokaisun@tmu.edu.cn; lehuixiao@nankai.edu.cn

Tables of content

Chemicals and materials……….……………………………………………..…S-4

Characterizations……………………..……………………….……………….S-4

Cancer cell membrane protein characterizations………………......…………..…….S-5

Measurement of photothermal effect……………………………………...…...…S-6

Cellular internalization……………………......…………………………..….S-7

Cytotoxicity assay………………………...…………………………..…….S-8

Hemolysis assay………………...………………..………..…………………S-8

Antiproliferative effect study *in vitro*……………..………………………….......S-9

In vitro MRI imaging……………………..…………….………………………S-10

Supporting figures………………………………………………………………S-12

References……………………………………..…………………..…….S-28

Methods

Chemicals and materials

Cetyltrimethyl ammonium bromide (CTAB), Rhodamine B (RhB) and dimethyl sulfoxide (DMSO) were purchased from Aladdin Reagent Co., Ltd. (Shanghai, China). Doxorubicin (DOX) was obtained from Ourchem Biotechnology Co., Ltd (Shanghai,China). Membrane protein extraction kit, Hoechst 33342, fluorescence dye DiO and propidium iodide (PI) were brought from Beyotime Biotechnology (Haimen, China). Protease inhibitor cocktail was obtained from Biotool LLC. (Shanghai, China). All other chemicals were purchased from Sinopharm Chemical Reagent Co., Ltd. (Shanghai, China) without further treatment.

Characterizations

Ultraviolet−visible (UV−vis) absorption spectra and fluorescence spectra were recorded by a spectrophotometer (UV−2450, Shimadzu, Japan) and a fluorescence spectrophotometer (F−4600, Hitachi, Japan) respectively. The morphological characterizations were characterized by a transmission electron microscopy (TEM, JEM2100, JEOL, Japan). High-resolution (HR) TEM imaging and elemental mapping were performed on FEI Talos F200X G2 (FEI, USA). Fourier transform infrared (FT−IR) spectra of the nanostrctures were recorded on a FT−IR spectrometer (AVATAR370, USA). X−ray photoelectron spectroscopy (XPS) measurements were performed on an Axis Ultra DLD photoelectron spectrometer (Kratos Analytical Ltd., U.K.). Raman spectra were measured by a LabRAM HR evolution microscope (Horiba Jobin Yvon) equipped with a 10x objective (NA = 0.25). All Raman

measurements were performed at 25 °C using a He-Ne laser with excitation power of 13.4 mW. The dynamic light scattering (DLS) results were determined by a Zetasizer Nano ZS system (Malvern, U.K.). The temperature elevation curve and thermal profiles of GNRs and GMNPs were obtained by a thermal imager (Fotric 226s) upon irradiation with an 808 nm laser at 1.5 W cm^{-2}. Confocal microscopic imaging experiments were performed on a laser confocal scanning microscope (LCSM, A1, Nikon, Japan). The T_1–MR phantom images and corresponding longitudinal (r_1) relaxation rate were recorded by a HT/MRSI60–60KY MRI system (1.2 T, Huantong, China). The optical density (OD) at 490 nm was measured by a microplate reader (Sunrise, Tecan, Switzerland). Flow cytometry analysis was performed on a BD FACSVerse flow cytometry (BD, USA).

Cancer cell membrane protein characterizations

A method according to previous work was used for the cell membrane proteins characterization [1-3]. First, the proteins from 4T1 cell lysate, 4T1 cell membrane fragments and 4T1 CM-GMNPs were separated by sodium dodecyl sulfate-polyacrylamide gel electrophoresis (SDS-PAGE). For western blotting analysis, the proteins were then transferred to polyvinylidene difluoride (PVDF) membranes. After being sealed with 10% nonfat milk in Tris buffered saline tween solution (50 mM Tris-HCl, pH=7.4; 150 mM NaCl; 0.1% Tween 20) for 1 h, the membranes were probed by diluted primary antibodies EpCAM (AF0141, Beyotime), N-cadherin (bs-1172R, Bioss), Na^+/K^+-ATPase (AF1864, Beyotime) as well as CD44 (AF1858, Beyotime), followed with horseradish-peroxidase-conjugated secondary

antibodies. At last, the films were cleaned and developed using a chemiluminescent substrate (Millipore). Corresponding protein bands were visualized on the Tanon−5200 Chemiluminescent Imaging System (Tanon Science and Technology). All experiments were repeated at least three times.

Measurement of photothermal effect

To evaluate the photothermal effect, different concentrations of GNRs and GMNPs solutions (7, 14, 21, 28 and 35 pM) were irradiated by 808 nm laser with a power density of 1.5 W cm^{-2} for 10 min. The photothermal images and corresponding temperature were recorded via an infrared thermal imaging instrument. In order to further determine the photothermal conversion efficiency of GNRs and GMNPs, these two samples (35 pM) were exposed to 808 nm laser until the temperature reached to a steady state. Then the laser was turned off and the solutions were cooled to room temperature. The photothermal conversion efficiency (η) was calculated according to Equation (1-4) [4]:

(1) $$\eta = \frac{hS(T_{max,NPs} - T_{max,solvent})}{I(1-10^{-A_{808}})}$$

(2) $$\tau_s = \frac{\mathrm{m}_D C_D}{hS}$$

(3) $$t = -\tau_s \, \mathrm{In}\theta$$

(4) $$\theta = \frac{T - T_{surr}}{T_{max,NPs} - T_{surr}}$$

where h is the heat transfer coefficient, S is the surface area of the container, $T_{max,NPs}$ and $T_{max,solvent}$ are maximum temperature of nanoparticles and water (GNRs: 53.4 ^{o}C; GMNPs: 50.2 ^{o}C; deionized H_2O: 30.6 ^{o}C). I is the incident laser power (1.5 W cm^{-2}), and A_{808} is the absorbance of GNRs and GMNPs at 808 nm (0.252 and 0.165

respectively). τ_s is the sample system time constant. m_D and C_D are the mass (1.0 g) and heat capacity (4.2 J g^{-1} ºC^{-1}) of the deionized water used as the solvent, respectively. θ is the dimensionless driving force temperature, T_{surr} is the ambient temperature of the surroundings (25.2 ºC), T is a temperature for solutions at a constant cooling time (t). θ is first obtained by eq (4), and τ_s of GNRs and GMNPs were then determined to 370.37 s by the linear fitting of t to –Inθ. η was then determined according to Equation (1).

Finally, the photostability of GNRs and GMNPs was also evaluated by a cyclic irradiation process. Specifically, the two samples (35 pM) were irradiated by 808 nm laser (1.5 W cm^{-2}) for 10 min and then cooled to room temperature without irradiation. Subsequently, additional four cycles were repeated. The temperatures of those solutions were recorded every 30 seconds.

Cellular internalization

For the cellular internalization assays, 4T1 cells were seeded in petri dishes and cultured in Dulbecco's Modified Eagle's medium (DMEM) containing 10% fetal bovine serum (FBS) and 1% penicillin-streptomycin at 37 °C for 24 h. To perform the targeting ability validation experiment, RhB absorbed-GMNPs (RhB-GMNPs) were first prepared by incubating GMNPs and RhB solution (1 mg mL^{-1}) under stirring at room temperature for 4 h, followed with centrifuging to remove excess RhB. The RhB-GMNPs were then extruded from 400 nm polycarbonate membranes for at least 5 times with 4T1 cell membrane fragments or HeLa cell membrane fragments. After being purified by centrifugation, the resulting CM_{4T1}-RhB-GMNPs and

CM_{HeLa}-RhB-GMNPs were co-incubated with as-prepared HeLa cells, 3T3 cells and 4T1 cells for 1 h at 37 °C. Finally, all cells were stained with Hoechst 33342 and were imaged under LCSM.

For the core-shell co-localization and cargo release assay, 4T1 cell membrane fragments were first stained with fluorescence dye DiO (0.02 mM) for 15 min at 4 °C, and were extruded from 400 nm polycarbonate membrane for at least 5 times with RhB-GMNPs. The DiO-labeled CM-RhB-GMNPs were then collected by centrifugation and were co-incubated with 4T1 cells for 1, 3 and 6 h, respectively. After stained with Hoechst for 25 min, the cells were photographed by LCSM.

Cytotoxicity assay

The cytotoxicity assays of GMNPs and CM-GMNPs were performed based on MTT ((3-(4,5-dimethylthiazol-2-yl)-2,5-diphenyltetrazolium bromide, a tetrazole)) test. In brief, HeLa cells were first seeded in 96-well plates with a density of 5×10^3 cells per well and cultured for 16 h. Afterwards, the cells were treated with the solution of GMNPs and CM_{4T1}-GMNPs with various concentration (0, 0.625, 1.25, 2.5, 5, 10, and 20 pM) for 24 h. Then 10 μL of MTT solution (5 mg mL^{-1}) was injected into each well and incubated with cells for another 4 h. Finally, the supernatant was discarded and the crystal on the bottom was dissolved with 150 μL of DMSO. The optical density (OD) at 490 nm was measured by a microplate reader.

Hemolysis assay

The fresh red blood cells (RBCs) used in the hemolysis assay were obtained from mice. The blood was diluted by physiological saline, and then RBCs were

isolated from serum by centrifugation (2000 rpm, 10 min). The RBCs were washed with physiological saline for five times. After carefully washing, RBCs were diluted with PBS solution. For the hemolysis assay, 0.2 mL of cell suspension was mixed with different samples. In detail, 0.8 mL of PBS was added as a negative control, 0.8 mL of water was added as a positive control, and 0.8 mL of product suspensions with varying concentration (1.25, 2.5, 5, 10, and 20 pM) were added as the experimental group. Finally, the mixtures were centrifuged at 10000 rpm for 10 min after co-incubation at 37 °C for 3 h. The absorbance of the upper supernatants was measured by UV–vis spectroscopy. The percentage hemolysis of the red blood cells was calculated by the following Equation (5):

$$(5)\ Hemolysis\ (\%) = \frac{A_{sample} - A_{control\,(-)}}{A_{control\,(+)} - A_{control\,(-)}}$$

where A_{sample}, $A_{control\,(-)}$, and $A_{control\,(+)}$ refer to the absorbance of experimental group, negative control group, and positive control group, respectively.

Antiproliferative effect study *in vitro*

The antiproliferative effect *in vitro* was first examined by MTT assay. Specifically, 4T1 cells were divided into three groups and seeded in 96-well plates with a density of 5×10^3 cells per well for 16 h. Then the cells were treated with CM-DOX-GMNPs and CM-GMNPs under different concentrations (0, 0.625, 1.25, 2.5, 5, 10, and 20 pM) for 24 h. After being washed with PBS solution and adding fresh medium, CM-GMNPs group and one of the CM-DOX-GMNPs groups were irradiated by 808 nm laser at 1.5 W cm^{-2} for 5 min. All cells were incubated for another 24 h. Finally, the cell viability was determined by MTT assay as described

above. In order to estimate the antiproliferative effect more intuitively, LCSM imaging was conducted. Similarly, 4T1 cells were divided into five groups and seeded in confocal culture dishes with a density of 8×10^4 to measure the antiproliferative effect *in vitro* using LCSM. After 24 h cultivation, all cells were replaced with fresh medium. Then CM-DOX-GMNPs solution (20 pM) was added into the group three and five while CM-GMNPs solution (20 pM) was added into group four. All cells were cultivated for 24 h. Subsequently, control group, CM-GMNPs group and one of the CM-DOX-GMNPs groups were irradiated by an 808 nm laser at 1.5 W cm^{-2} for 5 min and incubated for another 24 h. Finally, all cells were stained with Hoechst and PI to distinguish living and dead cells before LCSM imaging. Furthermore, flow cytometry analysis was also performed to estimate the antiproliferative effect of the nanostructure in this work. As described above, 4T1 cells were divided into three groups. CM-DOX-GMNPs with concentration of 60 pM were added in two of the three groups. The other group was added with cell culture medium. After co-cultured for 24 h, one of the groups added with CM-DOX-GMNPs was irradiated by an 808 nm laser at 1.5 W cm^{-2} for 5 min. Then, all cells were incubated for another 24 h. Finally, Annexin V-FITC/PI flow cytometry analysis was performed to investigate the cell apoptosis.

***In vitro* MRI imaging**

In vitro T1−MR phantom images and corresponding longitudinal time (T1) of GMNPs was performed by a HT/MRSI60–60KY MRI system (Huantong, shanghai). In detail, 1 mL GMNPs solution with different Mn^{2+} concentrations (0.2, 0.4, 0.6, 0.8

and 1.0 mM) in the presence and absence of GSH were prepared. The longitudinal relaxivity (r_1) was obtained by linear fitting of 1/T1 versus Mn^{2+} concentration. The relevant parameters were described as follow: TR/TE = 100.0/8.8 ms, matrix = 512 × 512, and slice thickness = 1 mm.

Supporting Figures

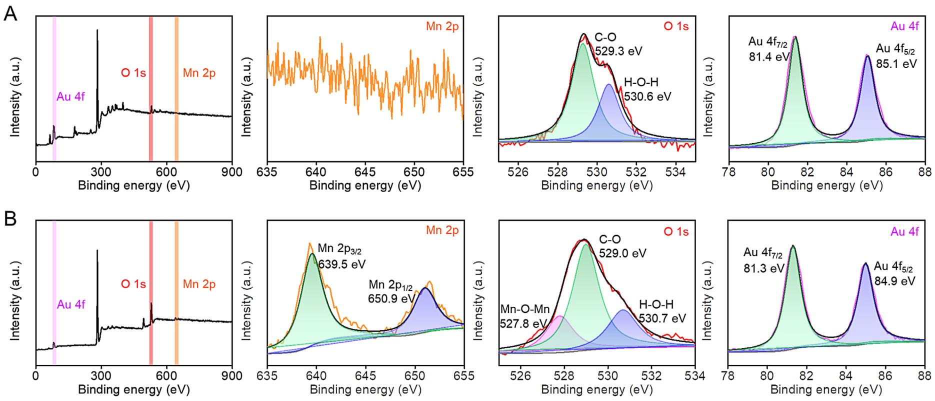

Figure S1. High resolution XPS spectra of GNRs (A) and GMNPs (B).

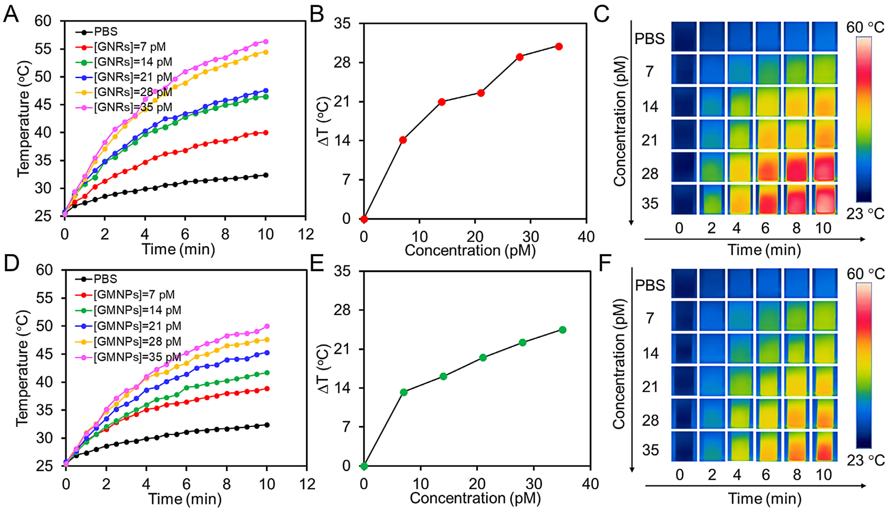

Figure S2. Temperature elevation cureves of different concentrations of GNRs (A) and GMNPs (D) upon 808 nm laser irradiation (1.5 W cm^{-2}). Plot of temperature change (ΔT) of GNRs (B) and GMNPs (E) over a period of 600 s versus different concentrations. The corresponding infrared thermal images of GNRs (C) and GMNPs (F) solution.

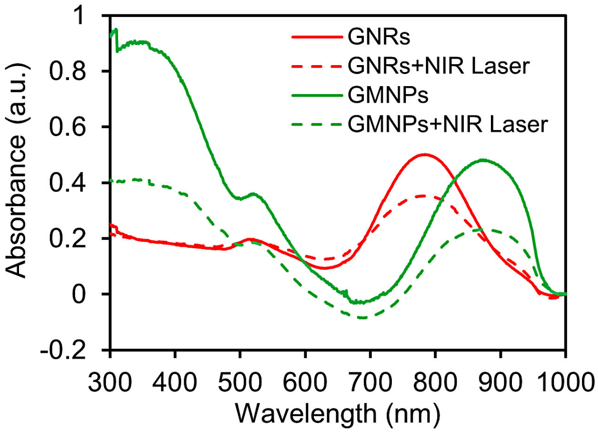

Figure S3. UV–vis absorption spectra of GNRs and GMNPs before and after laser irradiation (1.5 W cm^{-2}) for five cycles of heating and cooling.

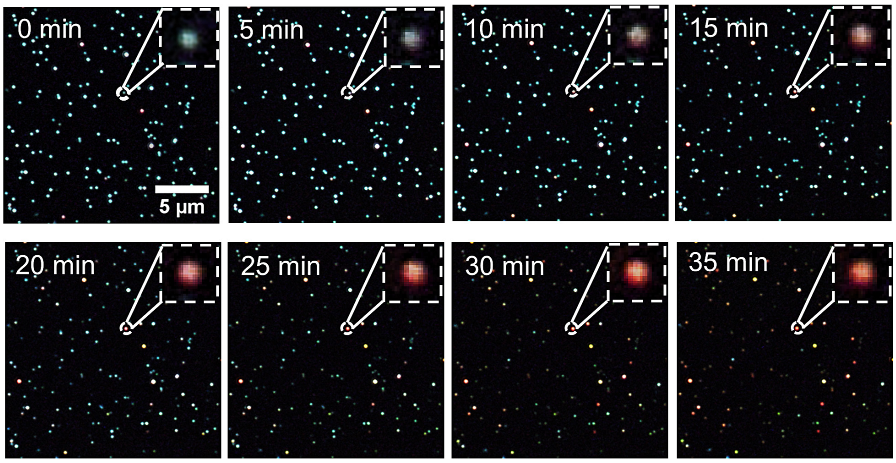

Figure S4. Dark-field optical microscopic images of GMNPs in the presence of 0.05 μM H_2O_2 (pH = 5.5) as a function of time.

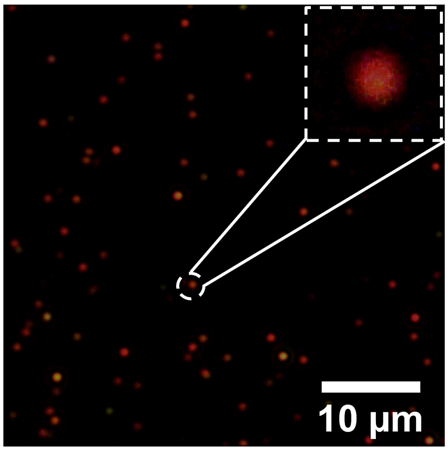

Figure S5. Representative dark-field optical microscopic image of GNRs.

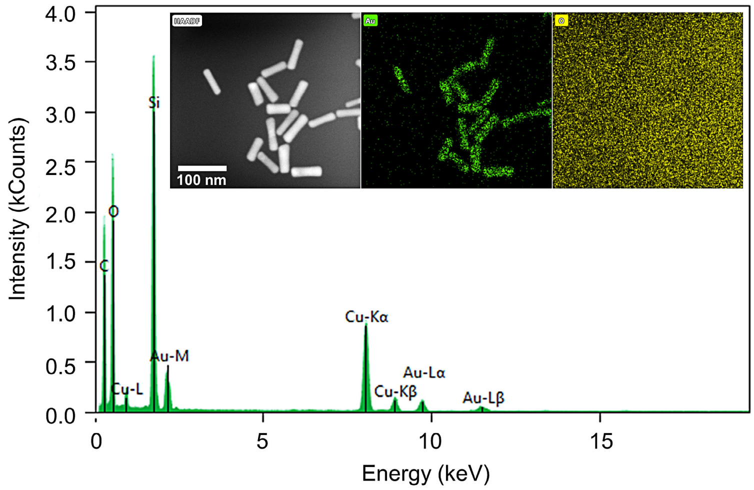

Figure S6. High resolution TEM image, energy dispersive spectroscopy spectrum, and elemental mapping analysis of GMNPs after etching by GSH.

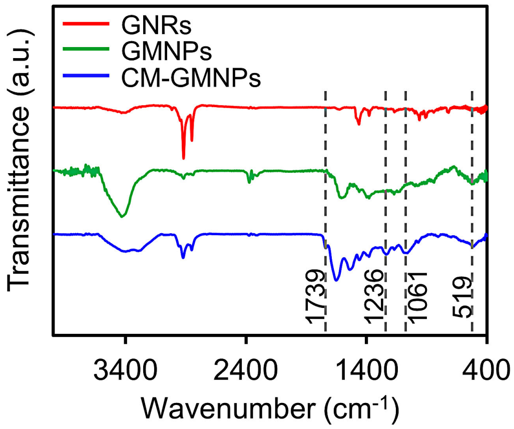

Figure S7. FT–IR spectra of GNRs (red line), GMNPs (green line) and CM-GMNPs (blue line). The peaks at 1061, 1236, and 1739 cm^{-1} in the spectrum of CM-GMNPs correspond to C–O–C stretching vibration, PO_2^- stretching vibration and C=O stretching vibration, respectively. The peak at 519 cm^{-1} in the spectra of GMNPs and CM-GMNPs is the stretching collision of O–Mn–O.

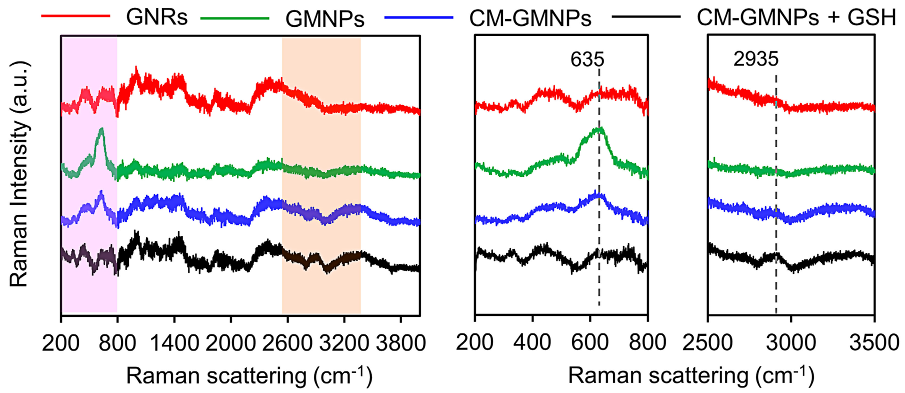

Figure S8. Raman spectra of GNRs (red line), GMNPs (green line), and CM-GMNPs before (blue line) and after (black line) etching with GSH. The peaks at 635 and 2935 cm^{-1} are assigned to the Mn–O stretching vibration and the superposition of C–H symmetric and asymmetric stretching vibrations, respectively.

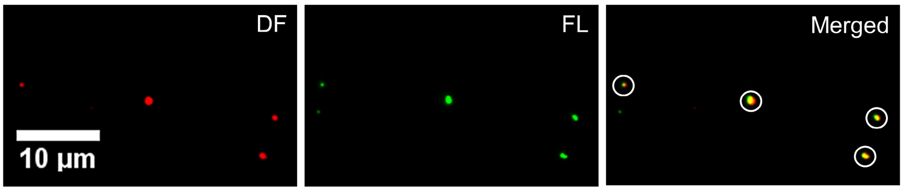

Figure S9. Colocalization analysis of the cell membrane and GMNPs. Representative dark-filed optical microscopic image of GMNPs (red), the corresponding fluorescence image of cell membrane stained with DiO (green), and the merged image of the GMNPs and cell membrane.

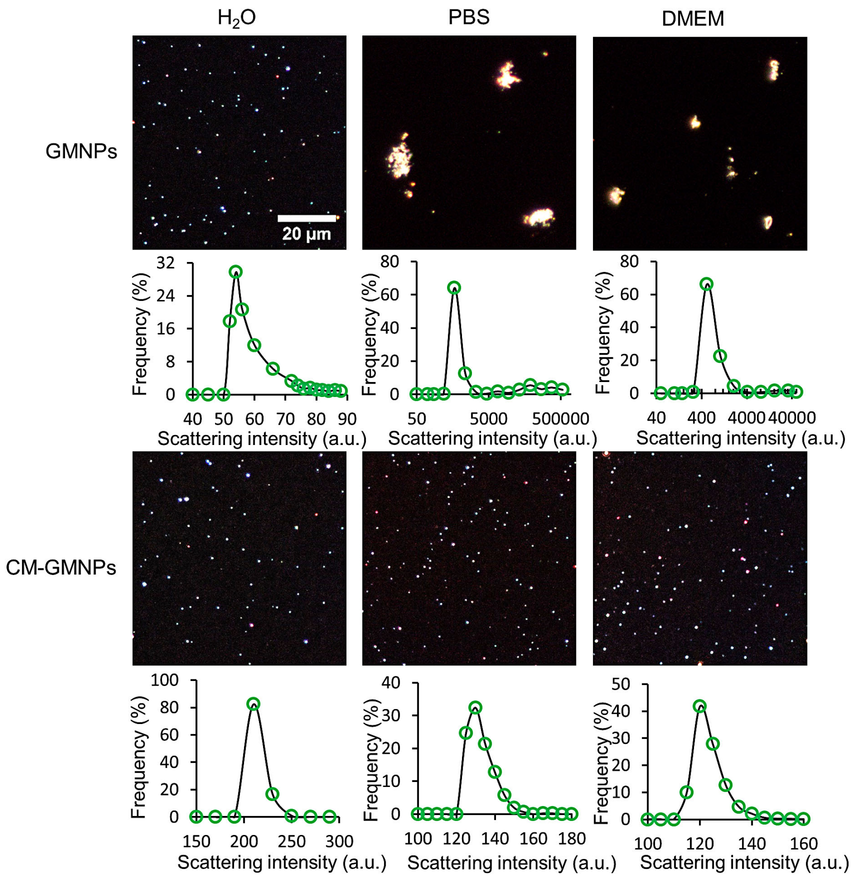

Figure S10. The statistical stability assays of GMNPs and CM-GMNPs in H_2O, PBS (10 mM, pH = 7.4) and DMEM.

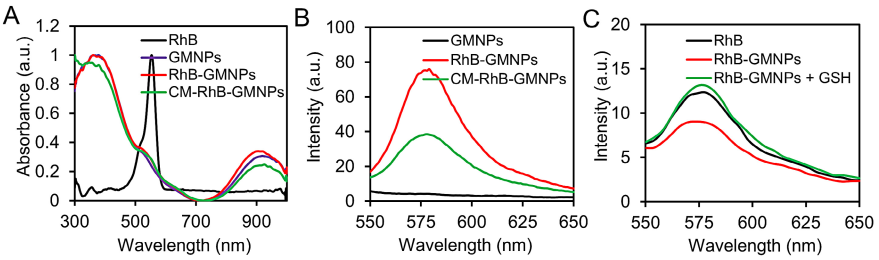

Figure S11. (A) UV–vis spectra of RhB, GMNPs, RhB-GMNPs and CM-RhB-GMNPs. (B) The fluorescence spectra of GMNPs, RhB-GMNPs and CM-RhB-GMNPs. (C) The fluorescence quenching and recovery of RhB-GMNPs in the absence and presence of GSH. (The concentration of RhB and GNRs are fixed at 0.1 μM and 110 fM, respectively)

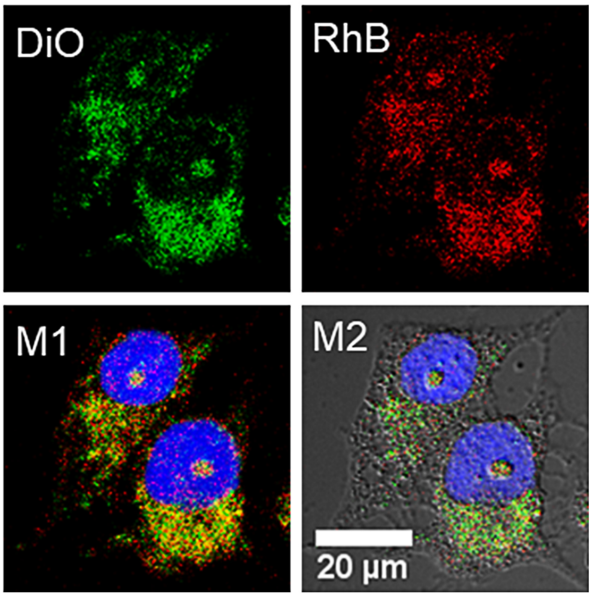

Figure S12. The LCSM images of cells incubated with DiO labeled CM-RhB-GMNPs. M1 is the overlay image of DiO channel and RhB channel. M2 is the overlay image of DiO channel, RhB channel and bright field.

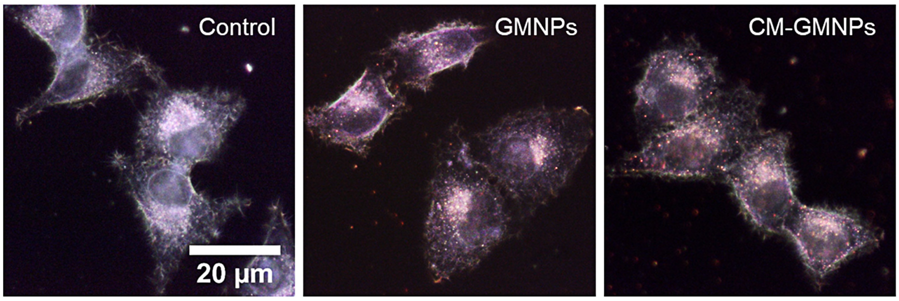

Figure S13. The dark-field optical microscopic images of HeLa cells treated with PBS (control), GMNPs and CM-GMNPs.

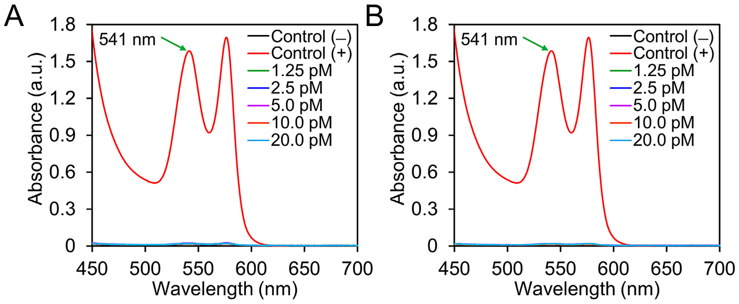

Figure S14. UV–vis absorption spectra of GMNPs (A) and CM-GMNPs (B) to red blood cells.

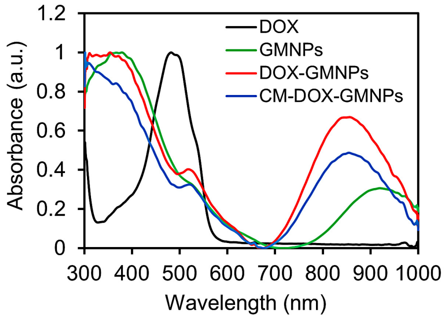

Figure S15. UV–vis spectra of DOX, GMNPs, DOX-GMNPs and CM-DOX-GMNPs.

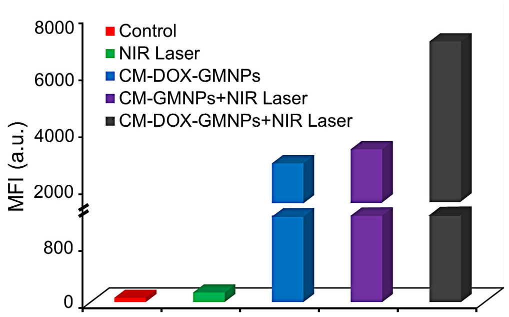

Figure S16. The corresponding mean fluorescence intensity of PI in Figure 3E.

References

1. Zhu JY, Zheng DW, Zhang MK, Yu WY, Qiu WX, Hu JJ, et al. Preferential cancer cell self-recognition and tumor self-targeting by coating nanoparticles with homotypic cancer cell membranes. Nano Lett. 2016; 16: 5895-901.
2. Chen Z, Zhao P, Luo Z, Zheng M, Tian H, Gong P, et al. Cancer cell membrane–biomimetic nanoparticles for homologous-targeting dual-modal imaging and photothermal therapy. ACS Nano. 2016; 10: 10049-57.
3. Xie Y, Zheng M, Chu X, Chen Y, Xu H, Wang J, et al. Paf1 and Ctr9 subcomplex formation is essential for Paf1 complex assembly and functional regulation. Nat Comm. 2018; 9: 3795.
4. Zhu H, Wang Y, Chen C, Ma M, Zeng J, Li S, et al. Monodisperse dual plasmonic Au@Cu_2-xE (E= S, Se) core@shell supraparticles: aqueous fabrication, multimodal imaging and tumor therapy at *in vivo* level. ACS Nano. 2017; 11: 8273-81.
